# Depressive disorders among physician parents in times of COVID-19 pandemic

**DOI:** 10.1192/j.eurpsy.2022.1414

**Published:** 2022-09-01

**Authors:** N. Regaieg, D. Ben Touhemi, A. Fayala, J. Boudabous, W. Kammoun, K. Khemakhem, I. Hadj Kacem, H. Ayadi, Y. Moalla

**Affiliations:** Hedi Chaker University Hospital, Child And Adolescent Psychiatry, Sfax, Tunisia

**Keywords:** physician parents, Covid-19, Depression

## Abstract

**Introduction:**

Studies have shown that physicians manifest a clear duty to work. For parents, reconciling work with parenthood is not easy and can even lead to depression.

**Objectives:**

To determine the prevalence and the factors for depression in Tunisian physician parents.

**Methods:**

This was a descriptive and analytical cross-sectional study of 93 Tunisian physician parents, conducted on google drive in March 2021, including a questionnaire containing the parents’ personal and professional data and the Beck Depression Inventory (BDI).

**Results:**

In our study, the sex ratio (M/F) was 0.05. The average age was 34.43 years old. Almost three-quarters of doctors (71.3%) were providing on duty services in the hospital while 69% were providing at least one call per month in COVID units. The average BDI score was 6.16. According to the BDI score, 60.9% of participants had depression. The BDI score was correlated with several types of dissatisfaction: dissatisfaction with the relationship with his child (p = 0.002), time devoted to the partner (0.001), time devoted to the child (p = 0.004), child’s educational style (p <0.001), time spent on leisure or personal activities (p <0.001), child’s academic performance (p = 0.001) and child’s behavior (p <0.001). Furthermore, the BDI score was associated with postponing having a child for career reasons (p = 0.038) and thinking that his career is slowed down by parenthood (p <0.001).

**Conclusions:**

Depression’s rate among physician parents appears to be significant. It’s associated with a feeling of guilt and dissatisfaction, hence the necessity of an early detection and management.

**Disclosure:**

No significant relationships.

